# The association between dietary inflammatory index and bone health in US adolescents: Analysis of the NHANES data

**DOI:** 10.1016/j.bonr.2024.101823

**Published:** 2025-01-03

**Authors:** Yuanyuan Zhang, Xuejing Wang, Shiguang Huo, Li Hong, Feifei Li

**Affiliations:** Department of pediatrics, Liaocheng Second People's Hospital, Liaocheng 252600, China

**Keywords:** Dietary inflammatory index, Bone health, Adolescents, Dual X-ray absorptiometry

## Abstract

**Introduction:**

Adolescents with a lower peak bone mineral density (BMD) and bone mineral content (BMC) have an elevated risk of osteoporosis in adulthood. The impact of diet on bone health, particularly its role in managing inflammation, which is a key factor in bone health, is gaining wider recognition. Despite evidence that anti-inflammatory diets can enhance bone health, the link between the dietary inflammatory index (DII) and bone health among US adolescents has not been thoroughly investigated. This study aimed to evaluate the correlation between DII score and bone health in this population.

**Methods:**

This cross-sectional study used data from the National Health and Nutrition Examination Survey (NHANES) of US adolescents aged 12–18 years, spanning surveys from 2001 to 2018. The DII was derived from dietary recall data obtained through questionnaire interviews. Bone health was assessed through total body less head (TBLH) BMD and BMC z-scores and lumbar spine bone mineral apparent density for age (BMAD_a_).

**Results:**

The study comprised 8773 adolescents with a mean age of 14.94 ± 1.97 years, 52.2 % were male. Multivariate linear regression analysis revealed a negative correlation between DII and lumbar spine BMAD_a_ (β = −0.000003, 95 % confidence interval [CI], −0.000005 to −0.000001; *P* = 0.001).This significant association remained robust when DII was treated as a categorical variable. Compared with individuals in quartile 1(Q1) DII scores (−3.71 to 1.04), those in Q4 (3.37 to 5.04) had lower BMAD_a_, with a regression coefficient of −0.00002 (95 % CI, −0.00003 to −0.000007, *P* < 0.001). DII was negatively correlated with TBLH BMC z-scores; however, the difference was not statistically significant. Subgroup analyses showed that DII was associated with lumbar spine BMAD_a_ and TBLH BMC z-scores in participants who were male, non-black, with a higher educational level, with a high family income, and underweight to normal weight. We found no significant association between DII and TBLH BMD z-scores.

**Conclusion:**

The findings from this cross-sectional analysis indicate a significant association between the DII and bone health among adolescents in the US, with a notable impact in males and non-black. These insights underscore the importance of adopting dietary patterns to mitigate inflammation and to support optimal bone health and metabolism.

## Highlights

• There is a negative relationship between dietary inflammatory index and bone health in adolescents in the United States, especially in the lumbar spine.
• There are racial and gender differences in this correlation, which is mainly observed in males and non-black individuals.
• The research underscores the importance of adopting anti-inflammatory dietary patterns to mitigate inflammation and support optimal bone health and metabolism in adolescents.

## 1 Introduction

Adolescents' bone health has emerged as a significant medical concern. Bone health refers to skeletal resistance to fractures, which is assessed by quantifying bone mineral reserves. This reserve is typically expressed in terms of bone mineral content (BMC) or bone mineral density (BMD) (Baronio and Baptista, 2022). Suboptimal bone mass and the incidence of fractures are prevalent not only among individuals with hereditary or acquired chronic conditions but also among healthy adolescents. A particularly vulnerable period is the peripubertal growth spurt, which is a critical phase in bone development (Cooper et al., 2004). Over the past several decades, there has been a documented increase in the prevalence of common childhood fractures, ranging from 35 % to 65 %. This trend is alarming and suggests that contemporary lifestyles may adversely affect the acquisition of early bone health (Khosla et al., 2003). Attaining a lower peak BMD in youth is considered a pivotal risk factor that can predispose individuals to osteoporosis in later years (Xue et al., 2020). Given that peak bone mass is typically reached around the age of 20, interventions aimed at enhancing bone health during this developmental window could be instrumental in mitigating the future risk of osteoporosis and related fractures.

Chronic low-grade inflammation, a common yet subtle condition, significantly affects health, particularly bone metabolism (Taheri et al., 2022). This pervasive inflammation is associated with an increased risk of osteoporosis, primarily due to the release of proinflammatory cytokines such as interleukin-1 (IL-1), interleukin-6 (IL-6), and tumor necrosis factor-alpha (TNF-α) (Lee et al., 2010; Wang and He, 2020). These cytokines are instrumental in osteoclastogenesis, the process of forming and activating osteoclasts, and in the cells responsible for bone resorption. An increase in osteoclast activity, if not balanced by bone formation, accelerates bone resorption, leading to net loss of bone density (Xiong et al., 2015). The impact of chronic inflammation is not limited to osteoclasts; it also disrupts the function of osteoblasts, which are responsible for bone formation. Osteoblasts, equipped with receptors for various cytokines, can be adversely affected by high levels of pro-inflammatory cytokines, impairing their ability to produce the bone matrix, thus exacerbating bone loss (Tanaka et al., 2005). One such factor is receptor activator of nuclear factor kappa-B ligand (RANKL), which is upregulated under inflammatory conditions, tipping the balance in favor of bone resorption. It also plays a pivotal role in osteoclast differentiation and function, including proliferation, multinucleation, activation, and survival (Fischer and Haffner-Luntzer, 2022; Rachner et al., 2011). Proinflammatory cytokines, notably TNF-α and IL-6, mediate osteocyte activity and influence both osteoblasts and osteoclasts. TNF-α has been shown to suppresses osteoblast activity at certain stages of differentiation, while IL-6 modulates the actions of these cells through intricate mechanisms (Wang and He, 2020). In summary, the multifaceted influence of chronic inflammation on bone metabolism involves dysregulation of both bone formation and resorption processes, with pro-inflammatory cytokines and the TNF family orchestrating these effects.

Numerous risk factors, including genetic and environmental susceptibility, have been proposed to increase the risk of osteoporosis and fractures. Dietary patterns and nutrient intake are modifiable non-pharmacological risk factors that improve bone health, and dietary inflammatory index (DII) is widely used to evaluate the inflammatory potential of a diet using a scoring algorithm for every dietary parameter and its effect on inflammatory biomarkers (Shivappa et al., 2014). Since the development of the current DII in 2014, many studies have investigated the association between the DII and a diverse range of chronic disease-related outcomes, including all-cause mortality, depression, and hypertension (Phillips et al., 2019; Hébert et al., 2019). Previous studies in Australian (Cervo et al., 2020), Chinese (Zhou et al., 2019), Korean (Na et al., 2019), American (Orchard et al., 2017), Iranian (Shivappa et al., 2016), and Mexican (Rivera-Paredez et al., 2022) populations have reported an association between DII and BMD, osteoporosis, and fracture risk; however, there is limited evidence on the association between DII and BMC in adolescents. We found that most of the evidence has been observed in postmenopausal women and adults, and different populations have reached different conclusions; therefore, it is necessary to study this association in people from different age groups. Thus, the aim of the present study was to evaluate the association between DII and bone health in adolescents in the US.

## 2 Materials and methods

### 2.1 Data source

The NHANES evaluates the health and nutritional status of non-institutionalized members of the US population using a stratified multistage probability sampling technique (Zipf et al., 2013). It is administered by the National Center for Health Statistics (NCHS) (Centers for Disease Control and Prevention, 2023). In this cross-sectional study, we extracted data from children and adolescents in the 2001–2018 NHANES cycles. In alignment with the guidelines of the International Society for Clinical Densitometry, we presented the BMC and BMD values for the lumbar spine and total body less head (TBLH) regions (Crabtree et al., 2014). Lumbar spine BMD and BMC data were derived from 3 NHANES cycles (2005–2006, 2007–2008 and 2009–2010), while TBLH data were obtained from 7 NHANES cycles (2001–2006 and 2011–2018). Data were analyzed between May 2024 and August 2024.

### 2.2 Standard protocol approval, registration, and patient consent

The NHANES was approved by the NCHS Ethics Review Committee in December 1998. The participants provided written informed consent prior to enrollment. The secondary analysis did not require additional Institutional Review Board approval (University of Connecticut, 2023).

### 2.3 Study design and population

**Fig. 1:**
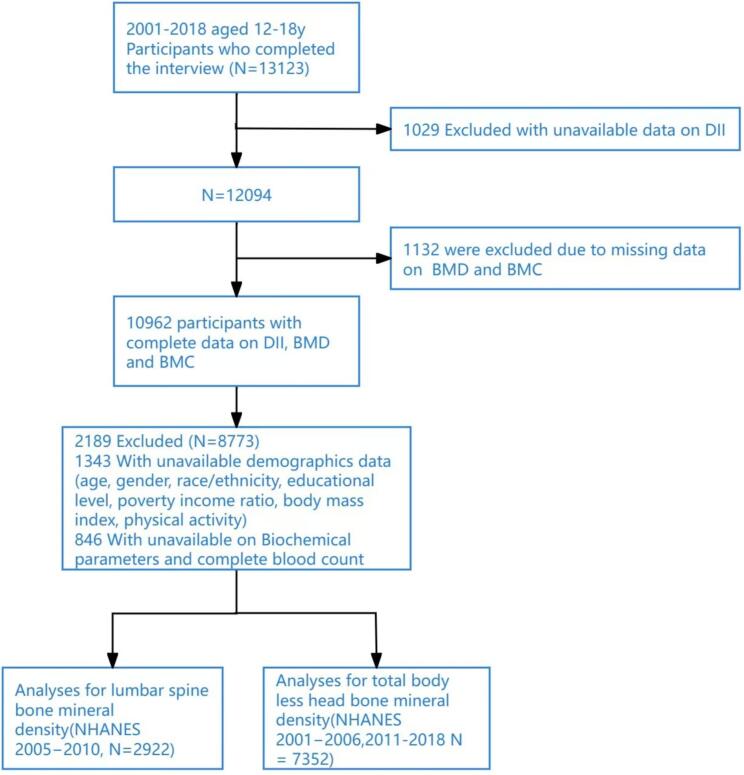
Inclusion and exclusion process for the final analysis was based on the 2001–2018 NHANES Survey. DII, dietary inflammation index; BMD, bone mineral density; BMC, bone mineral content; NHANES, National Health and Nutrition Examination Survey.

Of the 13,123 individuals aged 12–18 years who participated in the 2001–2018 NHANES, 1029 participants were excluded because of missing DII data; another 1132 adolescents with incomplete BMD and BMC data were excluded from our study. Subsequently, 2189 participants were excluded due to incomplete demographic data (*N* = 1343), which included age, gender, race/ethnicity, educational level, poverty income ratio (PIR), body mass index (BMI), and physical activity, as well as unavailable biochemical parameters and complete blood count (*N* = 846). This resulted in a final analytical sample of 8773 adolescents. In the NHANES, since only the 2005–2006, 2007–2008, and 2009–2010 cycles measured separate lumbar spine BMC and bone area, we conducted our study on lumbar spine BMAD_a_ using data from these three cycles, which included 2922 participants; for the study on TBLH BMD and BMC, we used data from the 7 cycles spanning 2001–2006 and 2011–2018, encompassing 7352 participants (Fig. 1).

### 2.4 Calculation of DII

The DII, introduced by Shivappa et al. in 2014 (Shivappa et al., 2014), serves as a metric for assessing the inflammatory potential inherent in an individual's dietary habits. This scoring mechanism is based on the examination of the influence of various dietary elements on a set of six biomarkers indicative of inflammation: IL-1β, IL-6, TNF-α, C-reactive protein (CRP), IL-4, and IL-10. Foods that are found to substantially elevate the concentrations of IL-1β, IL-6, TNF-α, or CRP, or to decrease those of IL-4 or IL-10, are assigned a positive score of +1, which denotes a pro-inflammatory impact. In contrast, items that exert an opposing influence earn a negative score of −1, reflecting an anti-inflammatory capacity. Foods that demonstrate no discernible effect on the aforementioned markers are granted a neutral score of 0.

The calculation of an individual's DII score is based on dietary intake data, which are typically obtained from the average of two 24-h dietary recalls (Liu et al., 2023; Liu et al., 2021). In dietary surveys, trained interviewers asked respondents about the types and amounts of food and beverages they have intaken in the past 24 h. Accurate nutritional values were calculated by the NHANES computer-assisted dietary interview system and the Automated Multiple Pass Method from 2001 to 2006 and 2011 to 2018, respectively (United States Department of Agriculture (USDA) and Agriculture Research Service FSRG, n.d.). For each nutrient, a *Z*-score was determined by taking the difference between the participant's total nutrient intake and the global average daily intake and then dividing by the nutrient's standard deviation (Shivappa et al., 2014). This *Z*-score was subsequently transformed into a percentile, which was doubled and then subtracted to arrive at the final DII score for that nutrient. Each food item's percentile value is then multiplied by its associated “inflammatory effect score” specific to the food parameter, resulting in the “food parameter-specific DII scores.” Finally, an individual's “overall DII score” was computed by aggregating these specific scores (Shivappa et al., 2014).

Although the 24-h recall method for dietary data collection has limitations in terms of reliability and accuracy, it provides a more nuanced view of the types and amounts of food consumed compared to food frequency questionnaires (Prentice et al., 2011; Subar et al., 2003). The DII has emerged as a respected metric for evaluating the inflammatory nature of an individual's diet, and its structural soundness and calculation process have been well documented in the literature (Shivappa et al., 2014).

In this study, a broad spectrum of dietary parameters was examined, encompassing a range of nutrients and components such as energy, protein, total fat, dietary fiber, carbohydrates, cholesterol, alcohol, and an array of vitamins including B12, B6, A, C, and E. Additionally, β-carotene, caffeine, monounsaturated fatty acids (MUFA), n-3 fatty acids, folic acid, and minerals such as iron (Fe), magnesium (Mg), niacin, riboflavin, polyunsaturated fatty acids (PUFA), saturated fats, selenium (Se), thiamine, and zinc (Zn) were examined. The participants were divided into quartiles based on their DII scores, facilitating a thorough examination of the correlation between dietary inflammatory potential and bone health outcomes.

### 2.5 Outcome: bone health

BMD and BMC measurements were conducted using dual-energy X-ray absorptiometry (DXA) by certified radiology professionals in the NHANES. Hologic QDR-4500A fan-beam densitometers (Hologic, Inc., Bedford, Massachusetts) were used in the 2001–2010 cycles; and Hologic Discovery model A densitometers (Hologic, Inc., Bedford, Massachusetts) were used in the 2011–2018 cycles. BMD and BMC scans from the 2001–2010 cycles were initially analyzed using Hologic Discovery v12.4 software and subsequently reanalyzed using Hologic APEX v3.0. For the 2011–2018 cycles, scans were analyzed using Hologic APEX v3.2 software and then reanalyzed using Hologic APEX v4.0. Further documentation is accessible online for those seeking a more in-depth understanding of the DXA scanning methodology documentation is accessible online.

In the assessment of growing children, it is crucial to appropriately adjust the results for spinal and TBLH BMC and BMD (Crabtree et al., 2014). To address this issue, we utilized bone mineral apparent density (BMAD), a widely recognized size-adjustment technique, which assumes a cubic shape for the vertebral body (Crabtree et al., 2013a; Kröger et al., 1993; Lu et al., 1996; Carter et al., 1992; Crabtree et al., 2013b). The BMAD calculation involves the use of a reference bone volume derived from the projected area (Ap) of the bone and an additional linear dimension. This linear dimension is presumed to be proportional to the average thickness of the bone within the scanned region (Katzman et al., 1991; Bachrach et al., 1999). In our analysis, we assumed that the lumbar spine regions L_1_-L_4_ to had cubic shapes. This approach allows for a more accurate representation of the bone density by considering the size and shape of the bone. To adjust for age-related variations in bone density, we applied an age-adjusted lumbar spine BMAD (BMAD_a_) (Crabtree et al., 2013b). BMAD_a_ provides a normalized measure of bone density specific to an individual's age, thereby enabling a more precise evaluation of bone health in the context of an individual's developmental stage. Consequently, we calculated BMADa values using the following formula (Carter et al., 1992; Katzman et al., 1991; Bachrach et al., 1999): lumbar spine BMAD_a_ = (BMC_a_/Ap^3/2^). BMC for age (BMC_a_) refers to the age-specific BMC *Z*-score (Zemel et al., 2011). For TBLH measurements, we applied an adjustment based on the Z-score, which was obtained from age-, sex-, race-, and height-specific reference curves representative of the US population. By using Z-scores, we standardized the TBLH BMC and BMD results, enabling meaningful comparisons across various age, sex, race, and height groups (Zemel et al., 2011).

### 2.6 Covariates

In the present study, demographics included age, sex, race/ethnicity (Mexican American, non-Hispanic white, non-Hispanic black, other Hispanics, and others), and educational level (less than high school, high school graduate or equivalent, above high school). The PIR is the ratio of family income to poverty guidelines appropriate for household size, representing the socioeconomic status of the participants. PIR was categorized as low (PIR < 1.3), medium (PIR = 1.3–3.5), and high (PIR > 3.5). In MEC (mobile examination center), the BMI was calculated based on the measured standing height (m) and weight (kg). Physical activity levels were evaluated using weekly metabolic equivalent task (MET) minute scores. According to NHANES protocols, weekly MET minutes were derived from the following equation: (8.0 METs × (weekly minutes of vigorous work-related activity + weekly minutes of vigorous leisure-time physical activity)) + (4.0 METs × (weekly minutes of moderate work-related activity + weekly minutes of moderate leisure-time physical activity + weekly minutes of walking or bicycling for transportation))(Yang et al., 2023).Laboratory data included white blood cell count (1000cells/μL), lymphocyte count (1000cells/μL), segmented neutrophil count (1000cells/μL), hemoglobin (g/dl), red cell distribution width, platelet count (1000cells/uL), serum CRP (mg/dL), alkaline phosphatase (ALP, IU/L), calcium(mmol/L), albumin(g/dl), and phosphorus(mmol/L). Relevant laboratory methods and detailed descriptions can be found on this website, http://www.cdc.gov/nchs/nhanes/.

### 2.7 Statistical analyses

Categorical variables were represented by proportions (%) while continuous variables were described as mean (standard deviation, SD) or median (interquartile range, IQR), as appropriate. Linear regression analyses (continuous variables) and χ^2^ tests (categorical variables) were used to compare variables between the groups. Linear regression analysis was used to explore the relationship between the DII and lumbar spine BMAD_a_, TBLH BMD z-scores, and BMC z-scores. TBLH BMD and BMC z-scores derived from age-, sex-, race-, and height-specific US national reference curves(Zemel et al., 2011). The DII was used as a categorical variable in the linear regression models, and a trend test was performed; the selection of confounders was based on clinical relevance and previous scientific literature. Two models were constructed: a crude model, which did not adjust for any covariates; and an adjusted model, adjusted for age, sex, race/ethnicity, education level, BMI, PIR, physical activity, white blood cell count, lymphocyte count, segmented neutrophil count, hemoglobin, red cell distribution width, platelet count, serum CRP, ALP, calcium, albumin, and phosphorus.

Subgroup analysis was performed to investigate the relationship between DII and lumbar spine BMAD_a_, TBLH BMD z-scores, and TBLH BMC z-scores based on participants' general characteristics. Heterogeneity among subgroups was assessed by including an interaction term in the model. Missing data, representing <5 % of the dataset, were addressed through listwise deletion for analysis. Furthermore, multiple imputations were utilized as a sensitivity analysis to ensure dataset completeness and mitigate potential bias resulting from missing values.

All statistical analyses were conducted using R Statistical Software (Version 4.2.2, http://www.R-project.org, The R Foundation) and Free Statistics Analysis Platform (Version 1.9.2, Beijing, China, http://www.clinicalscientists.cn/freestatistics). Free Statistics is a software tool that offers user-friendly interfaces for common analyses and data visualization. It leverages R as the statistical engine, with the graphical user interface (GUI) developed in Python. The platform enables reproducible analysis and interactive computation, with most analyses achievable in just a few clicks. A significance level of *P* < 0.05 (two-sided) was considered statistically significant.

## 3 Results

### 3.1 Baseline characteristics

After excluding records with incomplete data, the baseline characteristics of 8773 adolescents from the NHANES dataset spanning 2001–2018 were stratified into DII quartiles, as summarized in Table 1. The median age of the study population was 14.94 years, with a standard deviation of 1.97 years, and 52.2 % were male. Individuals with higher DII scores were more likely to be female, younger, of non-Hispanic Black ethnicity, have a higher level of education, exhibit a lower PIR, and have a higher BMI. Additionally, these individuals engaged less in physical activity, as indicated by their lower MET minutes per week. Health parameters also varied significantly across the DII quartiles. Notably, individuals in the higher DII quartiles had lower hemoglobin levels, higher red cell distribution width, elevated serum CRP levels, increased platelet counts, and decreased ALP and albumin levels. In terms of bone health, lumbar spine BMD, BMC, and BMAD_a_ showed significant differences across DII quartiles (*P* < 0.05). Specifically, lumbar spine BMD was the highest in the lowest DII quartile and showed a decreasing trend with increasing DII quartiles. BMC and BMAD_a_ followed this pattern, with the lowest values observed in the highest DII quartile. Conversely, TBLH BMD and BMC z-scores did not exhibit significant differences across the DII quartiles, suggesting that the relationship between the DII and bone health may be more pronounced in the lumbar spine region.

**Table 1:** Baseline characteristics of the study population by quartiles of DII in NHANES 2001–2018 cycles.

	Total	DII				P
		Quartile1 < 1.06	Quartile2 [1.06–2.39)	Quartile3 [2.39–3.36)	Quartile4 ≥ 3.36	
N	8773	2202	2194	2209	2168	
Age (years)	14.94 ± 1.97	15.12 ± 1.97	14.94 ± 1.97	14.83 ± 1.97	14.85 ± 1.98	< 0.001
Sex, n (%)						<0.001
Male	4578 (52.2)	1428 (64.9)	1240 (56.5)	1028 (46.5)	882 (40.7)	
Female	4195 (47.8)	774 (35.1)	954 (43.5)	1181 (53.5)	1286 (59.3)	
PIR group n (%)					<0.001	
<1.3	3488 (39.8)	809 (36.7)	854 (38.9)	900 (40.7)	925 (42.7)	
1.3–3.5	3320 (37.8)	833 (37.8)	825 (37.6)	827 (37.4)	835 (38.5)	
>3.5	1965 (22.4)	560 (25.4)	515 (23.5)	482 (21.8)	408 (18.8)	
Race/ethnicity, n (%)						<0.001
Mexican American	2399 (27.3)	652 (29.6)	661 (30.1)	575 (26)	511 (23.6)	
Non-Hispanic White	2577 (29.4)	657 (29.8)	645 (29.4)	627 (28.4)	648 (29.9)	
Non-Hispanic Black	2476 (28.2)	496 (22.5)	597 (27.2)	677 (30.6)	706 (32.6)	
Other Hispanic	633 (7.2)	177 (8)	139 (6.3)	174 (7.9)	143 (6.6)	
Other	688 (7.8)	220 (10)	152 (6.9)	156 (7.1)	160 (7.4)	
Education level, n(%)						<0.001
Less than high school	2654 (30.3)	623 (28.3)	670 (30.5)	667 (30.2)	694 (32)	
High school graduate or equivalent	2127 (24.2)	478 (21.7)	522 (23.8)	554 (25.1)	573 (26.4)	
Above high school	3992 (45.5)	1101 (50)	1002 (45.7)	988 (44.7)	901 (41.6)	
BMI (kg/m2)	23.77 ± 5.86	23.11 ± 5.42	23.60 ± 5.73	23.95 ± 6.09	24.44 ± 6.12	< 0.001
MET minutes per week	1761.38 ± 3295.23	2114.54 ± 3650.14	1744.25 ± 3410.38	1522.90 ± 2893.30	1663.02 ± 3150.88	< 0.001
Lumbar spine BMD (g/cm^2^)*	0.94 ± 0.16	0.951 ± 0.16	0.929 ± 0.16	0.938 ± 0.16	0.949 ± 0.16	0.022
Lumbar spine BMC (g)*	53.90 ± 14.20	56.68 ± 15.25	53.11 ± 14.26	52.95 ± 13.83	52.87 ± 13.01	< 0.001
Lumbar spine BMAD_a_(g/cm^3^)*	0.00002 ± 0.0001	0.00003 ± 0.00009	0.00001 ± 0.0001	0.00002 ± 0.0001	0.00002 ± 0.0001	0.041
TBLH BMD z-score**	0.14 ± 0.08	0.15 ± 0.07	0.15 ± 0.08	0.14 ± 0.08	0.14 ± 0.08	0.112
TBLH BMC z-scores**	0.21 ± 0.10	0.21 ± 0.09	0.22 ± 0.10	0.21 ± 0.11	0.21 ± 0.10	0.129
**Biochemical parameters**						
White blood cell (1000cells/μL)	6.94 ± 2.04	6.92 ± 1.97	6.95 ± 2.06	6.99 ± 2.08	6.90 ± 2.07	0.515
Lymphocyte (1000cells/μL)	2.26 ± 0.64	2.26 ± 0.62	2.25 ± 0.65	2.28 ± 0.64	2.26 ± 0.63	0.427
Segmented neutrophils (1000cells/μL)	3.87 ± 1.70	3.84 ± 1.63	3.89 ± 1.71	3.90 ± 1.72	3.84 ± 1.73	0.595
Hemoglobin(g/dl)	14.03 ± 1.35	14.31 ± 1.38	14.13 ± 1.31	13.91 ± 1.33	13.75 ± 1.31	< 0.001
Red cell distribution width	12.77 ± 1.03	12.73 ± 0.96	12.72 ± 0.94	12.77 ± 1.08	12.84 ± 1.12	< 0.001
Platelet count(1000cells/uL)	275.21 ± 64.63	265.68 ± 61.67	273.41 ± 63.57	280.45 ± 65.14	281.39 ± 66.86	< 0.001
Serum CRP (mg/dL), (Median (IQR))	0.050(0.020, 0.170)	0.050 (0.020, 0.140)	0.050 (0.020, 0.150)	0.051 (0.020, 0.180)	0.056 (0.020, 0.180)	0.002
ALP (IU/L)	148.95 ± 98.97	152.42 ± 98.37	152.21 ± 98.55	149.88 ± 101.93	141.19 ± 96.56	< 0.001
Albumin(g/dl)	4.42 ± 0.30	4.47 ± 0.30	4.43 ± 0.30	4.39 ± 0.29	4.38 ± 0.29	< 0.001
Phosphorus(mmol/L)	1.43 ± 0.22	1.44 ± 0.22	1.44 ± 0.22	1.44 ± 0.22	1.42 ± 0.21	< 0.001
Calcium(mmol/L)	2.42 ± 0.08	2.42 ± 0.08	2.42 ± 0.08	2.42 ± 0.08	2.41 ± 0.08 < 0.001	

DII, dietary inflammation index; PIR, poverty income ratio; BMI, body mass index; MET, metabolic equivalent task; BMD, bone mineral density; BMC, bone mineral content; BMAD_a_, bone mineral apparent density for age; TBLH, total body less head; CRP, C-reactive protein; IQR, interquartile range; ALP, alkaline phosphatase.* data were obtained from the NHANES 2005–2010 cycles; **data were obtained from the NHANES 2001–2006 and 2011–2018 cycles.

### 3.2 Association of DII and lumbar spine BMAD_a_

The findings from the multivariable linear regression analysis examining the relationship between the DII and lumbar spine BMAD_a_ are displayed in Table 2. The table showcases outcomes for both the unadjusted (crude) model and the adjusted model. The latter accounts for various factors including age, sex, race/ethnicity, BMI, PIR, education level, physical activity, white blood cell count, lymphocyte count, segmented neutrophil count, hemoglobin, red cell distribution width, platelet count, serum CRP, ALP, calcium, albumin, phosphorus, and NHANES cycle.

**Table 2:** Association between DII and lumbar spine BMAD_a_, TBLH BMD z-Scores and TBLH BMC z-Scores.

Variable	Crude model		Adjusted model	
	β(95 % CI)	P value	β(95 % CI)	P value
**Lumbar spine BMAD**_**a**_^**⁎**^				
DII	−0.000002(−0.000004–0)	0.087	−0.000003(−0.000005 ~ −0.000001)	0.001
Q1	0(Ref)		0(Ref)	
Q2	−0.00002 (−0.00003 ~ −0.000005)	0.005	−0.00002 (−0.00003 ~ −0.00001)	<0.001
Q3	−0.000009 (−0.00002–0.000001)	0.092	−0.00002 (−0.00002 ~ −0.000006)	<0.001
Q4	−0.00001(−0.00002–0.000001)	0.064	−0.00002 (−0.00003 ~ −0.000007)	<0.001
P for trend		0.162		<0.001
**TBLH BMD z-Scores**^**⁎⁎**^				
DII	−0.0011 (−0.0022 ~ −0.00003)	0.044	0.0005 (−0.0004–0.0015)	0.240
Q1	0(Ref)		0(Ref)	
Q2	−0.0006 (−0.0055–0.0044)	0.826	0.0017 (−0.0024–0.0057)	0.415
Q3	−0.0039 (−0.0089–0.0011)	0.126	0.0002 (−0.0039–0.0043)	0.916
Q4	−0.0045 (−0.0095–0.0005)	0.077	0.0024 (−0.0018–0.0065)	0.264
P for trend		0.037		0.400
**TBLH BMC z-Scores**^**⁎⁎**^				
DII	−0.0003 (−0.0017–0.0011)	0.632	−0.0002 (−0.0013–0.0009)	0.701
Q1	0(Ref)		0(Ref)	
Q2	0.0036 (−0.0029–0.01)	0.282	0.0012 (−0.0036–0.0060)	0.674
Q3	0.00009 (−0.0064–0.0065)	0.979	−0.0013 (−0.0061–0.0036)	0.604
Q4	−0.0026 (−0.0091–0.0038)	0.428	−0.0003 (−0.0052–0.0046)	0.903
P for trend		0.278		0.670

Crude model: no covariates were adjusted.

Adjusted model was adjusted for age, sex, race/ethnicity, education level, BMI, PIR, physical activity, white blood cell count, lymphocyte count, segmented neutrophils count, red cell distribution width, hemoglobin, platelet count, serum CRP, ALP, calcium, albumin, phosphorus, NHANES cycle.

DII, dietary inflammation index; BMAD_a_, Bone mineral apparent density for age; TBLH, total body less head; BMD, bone mineral density; BMC, bone mineral content; NHANES, National Health and Nutrition Examination Survey; BMI, body mass index; PIR, poverty income ratio; CI, confidence interval; Ref, reference; CRP, C-reactive protein; ALP, alkaline phosphatase. *data were obtained from the NHANES 2005–2010 cycles; **data were obtained from the NHANES 2001–2006, 2011–2018 cycles.

A high DII score was associated with lower BMAD_a_ (β = −0.000003(95 % CI: −0.000005 to −0.000001; *P* = 0.001), after adjusting for potential confounders. This association was maintained when the DII scores were transformed into categorical variables as quartiles. Compared with individuals in quartile 1(Q1) DII scores (−3.71 1.04), those in Q4 (3.37 to 5.04) had lower BMAD_a_, with a regression coefficient of −0.00002 (95 % CI: −0.00003 to −0.000007, *P* < 0.001).

**Fig. 2:**
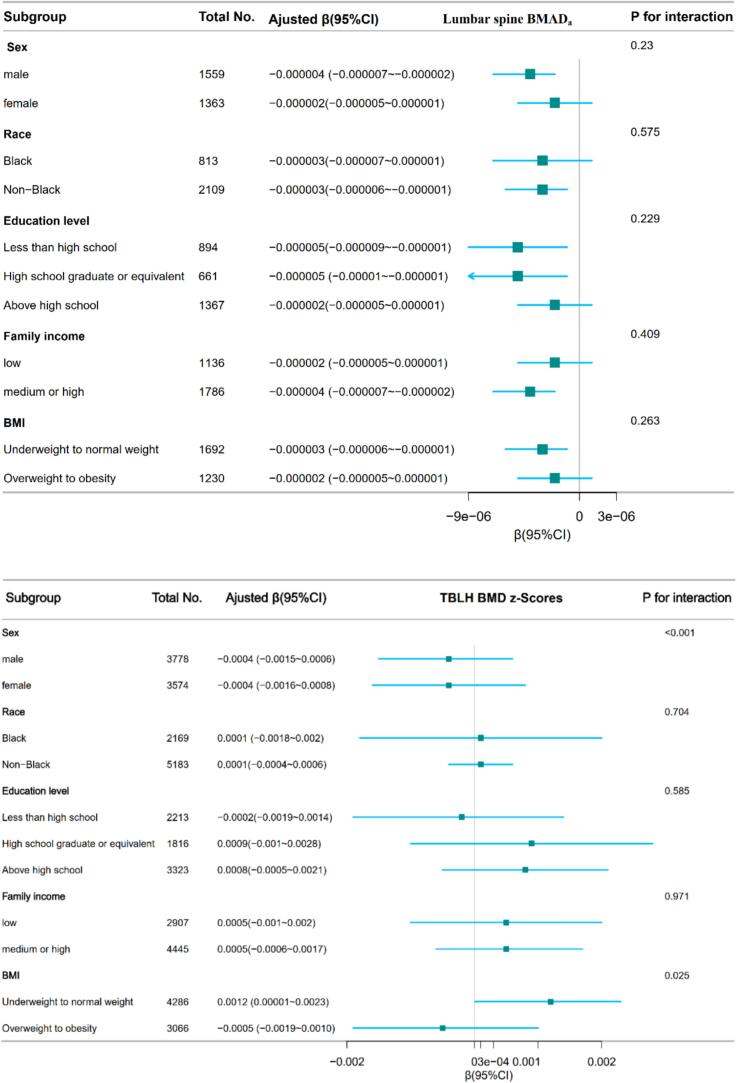

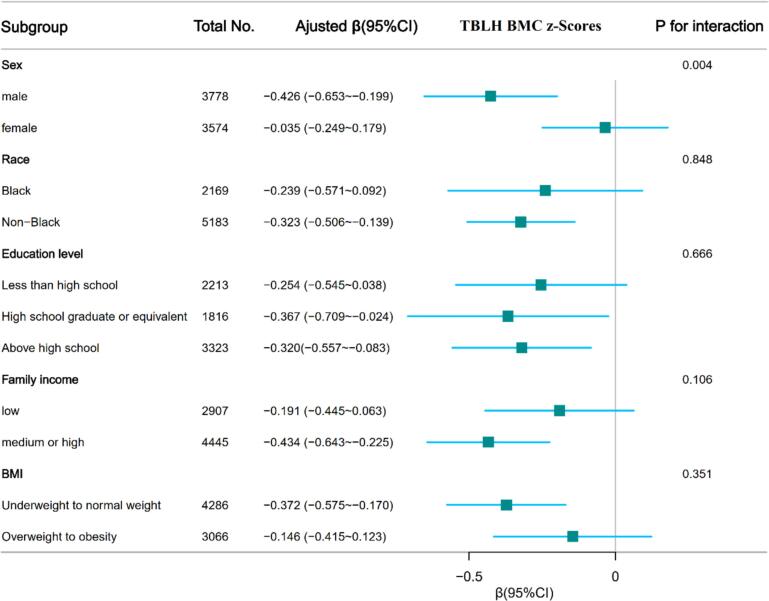
Association between DII and lumbar spine BMAD_a_, TBLH BMD z-scores and TBLH BMC z-scores according to the general characteristics. Except for the stratification factor itself, the stratifications were adjusted for all variables (age, gender, race/ethnicity, education level, BMI, PIR, physical activity, white blood cell count, lymphocyte count, segmented neutrophils count, red cell distribution width, hemoglobin, platelet count, serum CRP, ALP, calcium, albumin, phosphorus, NHANES cycle). DII, dietary inflammation index; BMAD_a_, Bone mineral apparent density for age; TBLH, total body less head; BMD, bone mineral density; BMC, bone mineral content; NHANES, National Health and Nutrition Examination Survey; BMI, body mass index; PIR, poverty income ratio; CI, confidence interval; CRP, C-reactive protein; ALP, alkaline phosphatase. P for interaction: The *P* value associated with the interaction term in the multivariate regression model, assessing the significance of the interaction effect between the variables on sex, race, education, BMI, and PIR level.

We conducted stratified analyses to assess potential effect modification by sex (male, female), race (black, non-black), educational level (less than high school, high school graduate or equivalent, above high school), family income (low, medium or high), and BMI (underweight to normal weight, overweight to obese) (Fig. 2). DII was negatively associated with BMAD_a_ in participants who were males (β = −0.000004, 95%CI:-0.000007 ~ −0.000002, *P* = 0.002), non-black (β = −0.000003, 95%CI: −0.000006 ~ −0.000001, *P* = 0.004), with a higher educational level (β = −0.000005, 95%CI: −0.00001 ~ −0.000001, *P* = 0.02), with a high family income (β = −0.000004, 95%CI: −0.000007 ~ −0.000002, *P* = 0.0006) and with underweight to normal weight (β = −0.000003, 95%CI: −0.000006 ~ −0.000001, *P* = 0.017).

### 3.3 Association of DII and TBLH BMD z-scores and TBLH BMC z-scores

Table 2 presents the results of the multivariable linear regression analysis examining the association between DII and TBLH BMD and BMC z-scores. In the crude model, DII was negatively correlated with TBLH BMD z-scores (β = −0.0011, 95 % CI: −0.0022 ~ −0.00003, *P* = 0.044). However, this negative correlation was no longer significant when adjusted for potential confounders. DII was negatively correlated with TBLH BMC z-scores, but the difference was not statistically significant.

Fig. 2 presents the results of subgroup analyses. DII was negatively associated with TBLH BMC z-scores in participants who were males (β = −0.426, 95 % CI:-0.653 ~ −0.199, *P* = 0.0002), non-black (β = −0.323, 95 % CI: −0.506 ~ −0.139, *P* = 0.0006), with a higher educational level (β = −0.320, 95 % CI: −0.557 ~ −0.083, *P* = 0.008), with a high family income (β = −0.434, 95 % CI: −0.643 ~ −0.225, *P* < 0.001) and with underweight to normal weight (β = −0.372, 95 % CI: −0.575 ~ −0.170, *P* = 0.0003). There is sex-based interaction effect between DII and TBLH BMD z-scores and TBLH BMC z-scores (*P* < 0.05). Despite P < 0.05, for the interaction of BMI and sex in the association between DII and TBLH BMD z-scores, the findings may not be clinically significant considering the multiple testing and similar directionality of the associations.

To evaluate the robustness of our results, we utilized multiple imputation methods to fill in missing covariate date. The results of these sensitivity analyses (Supplementary Table S1) align with our primary findings.

## 4 Discussion

Based on a comprehensive cross-sectional study conducted among adolescents in the United States, our study revealed a negative association between DII and lumbar spine BMAD_a_. Moreover, we found that the relationship between the DII and bone health was affected by sex and ethnicity. The association is also influenced by socioeconomic status and body weight, as they are known to affect dietary habits and physical activity levels, both of which can have a significant impact on bone density and overall skeletal health.

To our knowledge, this is the first study to demonstrate a negative relationship between the DII and BMD and BMC in healthy adolescents in the United States. A meta-analysis showed that a high DII score can affect bone health in adult (Taheri et al., 2022). However, existing research on adolescents in the United States has yielded inconsistent results, some studies reporting no significant correlation between the DII and bone turnover markers (Coheley et al., 2019). In contrast to previous studies, the present study analyzed data from the NHANES after adjusting for potential confounders using multivariable regression analysis to make the results generalizable to the adolescent population in the US. Dose-response analysis revealed a linear relationship between DII and bone health especially in the lumbar spine. This may be related to the deceleration of limb growth and the acceleration of trunk growth during puberty (Seeman, 1998). Additionally, the association between DII and bone health remained stable in sensitivity analysis and subgroup analyses.

Another noteworthy finding of the current study was that a significant inverse relationship was observed, mainly in males. Previous studies conducted in populations aged ≥50 years and those with chronic kidney disease have indicated that higher DII scores are associated with an increased risk of osteoporosis in women (Zhao et al., 2022; Meng et al., 2023). Another study showed that inhalation exposure to lipopolysaccharide, which can induce airway inflammation, leads to BMD deterioration only in male mice (not females) (Nelson et al., 2018). DII score was not correlated with bone health in female collegiate athletes (Gieng et al., 2024); in a study of the association between lumbar spine BMD and platelet count, this association was observed only in males (Zhang et al., 2024). Evidence indicates that the relationship between inflammation and bone health appears to be sex specific in different populations. The biological underpinnings of this susceptibility are multifaceted, and can be attributed to a combination of hormonal, genetic, and lifestyle factors. Hormonally, animal studies have indicated that male rodents that have undergone surgical castration have profound decreases in BMD and predicted bone strength (Ryu et al., 2016), indicating that androgens have anabolic effects on the bone. However, estradiol is considered to have a greater impact on bone protection during the prepubertal and pubertal periods (Falahati-Nini et al., 2000). During menopause, an increase in circulating IL-1 and TNF directly affects osteoclasts to increase bone resorption, and treatment with inhibitors of these immune factors mitigates the increase in bone turnover markers (Charatcharoenwitthaya et al., 2007). Estrogens in females have been shown to exert anti-inflammatory effects, which might protect against the detrimental effects of a high-DII diet to some extent. However, the precise mechanisms involved have not been fully elucidated. Genetic inheritance has been shown to account for 50–85 % of the variance in peak bone mass in both mouse and human studies (McGuigan et al., 2002; Beamer et al., 1996). Genetic factors play a pivotal role in determining the rate of growth plate expansion and elongation of long bones in the developing skeleton (Parfitt, 1997). During male puberty, there is an increase in periosteal expansion, which contributes to a greater bone size than in females. This period of rapid growth and development is a critical window for establishing bone health, which, in turn, is more susceptible to the effects of inflammation and other factors (Parfitt, 1994). Lifestyle factors including physical activity and dietary habits also contributed to the observed differences. Males, who traditionally have higher muscle mass and engage in more physically demanding activities, may have increased bone turnover, which may be more susceptible to the effects of inflammation (McGuigan et al., 2002; Parfitt, 1994). In conclusion, the impact of DII on bone health, with a noted gender difference favoring increased susceptibility in males, is a complex interplay of hormonal, genetic, and lifestyle factors. Further research is necessary to fully dissect these interactions and to develop targeted interventions for the prevention and treatment of bone disorders related to inflammatory diets.

Our investigation demonstrated that the influence of DII on BMD was more notable in individuals of non-black descent, underscoring racial inequalities. Previous studies have suggested that there are no racial or sex disparities in the BMAD of the trabecular bone in the vertebral body before puberty, and race-based divergences have become apparent during puberty (Gilsanz et al., 1998). A study in adults examining the correlation between bone density and platelet count found a significant relationship predominantly in Non-Hispanic Whites (Zhang et al., 2024). Additionally, reference standards for BMD and BMC in children were established considering age, sex, and race (Zemel et al., 2011), confirming the necessity of considering racial variations, particularly during puberty, when assessing changes in bone density and its determinants in children. The specific mechanisms underlying the observed impact of DII on bone density exclusively in non-black individuals in our study are complex and may involve growth patterns, hormone levels, and sensitivity (Seeman, 1998). The specific mechanisms still require further investigation.

The present study has certain limitations. First, this study was conducted in a US adolescent population; additional research is required to confirm whether our results can be generalized to other populations. Second, the residual confounding effects could not be excluded. We constructed multivariable linear regression models and performed subgroup and sensitivity analyses to control for the effects of potential confounders on the relationship between DII score and bone health. Third, because this was a cross-sectional study, the causality of the relationship between the DII and bone health could not be determined. Therefore, longitudinal studies are required to determine whether the observed relationship between the DII and bone health is causal.

The findings of this study contribute to the growing body of evidence linking dietary patterns to bone health outcomes. The inverse relationship observed between DII scores and bone health indicators suggests that diets with higher inflammatory potential may have detrimental effects on bone health in adolescents in the US. These findings underscore the importance of promoting anti-inflammatory dietary patterns rich in fruits, vegetables, whole grains, and healthy fats to support optimal bone health. Future longitudinal studies are needed to establish a causal relationship between the DII and bone health outcomes, informing targeted interventions to improve bone health in the US population.

## 5 Conclusion

In conclusion, this cross-sectional study provides evidence of an inverse association between DII scores and bone health indicators in adolescents in the US, with racial and sex differences. These results suggest that dietary patterns with higher inflammatory potential may have a negative impact on bone health. Promoting anti-inflammatory diets could be a promising strategy to enhance bone health and reduce the risk of osteoporosis in the US population. Future longitudinal studies are needed to establish a causal relationship between the DII and bone health outcomes, informing targeted interventions to improve bone health in adolescents in the US.

The following is the supplementary data related to this article.

Supplementary Table S1 Association between DII and lumbar spine BMAD_a_, TBLH BMD z-Scores and TBLH BMC z-Scores after multiple imputation. Supplementary Table S1

## CRediT authorship contribution statement

**Yuanyuan Zhang:** Writing – review & editing, Writing – original draft, Formal analysis, Conceptualization. **Xuejing Wang:** Software, Data curation. **Shiguang Huo:** Software. **Li Hong:** Validation, Supervision. **Feifei Li:** Writing – review & editing, Supervision, Project administration.

## Funding sources

This research did not receive any specific grant from funding agencies in the public, commercial, or not-for-profit sectors.

## Declaration of competing interest

The authors declare that they have no known competing financial interests or personal relationships that could have appeared to influence the work reported in this paper.

## Data Availability

Data will be made available on request.
