# Supplementary material for: The association between dietary inflammatory index and bone health in US adolescents: Analysis of the NHANES data
Source: Bone Rep. 2025 Jan 3;24:101823. doi: 10.1016/j.bonr.2024.101823 (PMC11758120; doi:10.1016/j.bonr.2024.101823)
Supplement: Supplementary Table S1 — Association between DII and lumbar spine BMADa, TBLH BMD z-Scores and TBLH BMC z-Scores after multiple imputation. [file mmc1.docx]

**Supplementary Table S1** Association between DII and lumbar spine BMAD_a_, TBLH BMD z-Scores and TBLH BMC z-Scores after multiple imputation.

|  | Crude model | |  | Adjusted model | |
| --- | --- | --- | --- | --- | --- |
| Variable | β(95% CI) | P value |  | β(95% CI) | P value |
| **Lumbar spine BMADa*** |  |  |  |  |  |
| DII | -0.000001(-0.000003~0.000001) | 0.18 |  | -0.000003(-0.000005~-0.000001) | 0.002 |
| Q1 | 0(Ref) |  |  | 0(Ref) |  |
| Q2 | -0.00001(-0.00002~-0.000005) | 0.004 |  | -0.00002(-0.00003~-0.00001) | <0.001 |
| Q3 | -0.00001(-0.00002~0) | 0.051 |  | -0.000016 (-0.000025~-0.000007) | <0.001 |
| Q4 | -0.000008(-0.00002~0.000002) | 0.115 |  | -0.000015(-0.000024~-0.000007) | <0.001 |
|  |  | 0.232 |  |  | 0.004 |
| **TBLH BMD z-Scores^**^** |  |  |  |  |  |
| DII | -0.0011 (-0.0020~-0.0001) | 0.031 |  | 0.0006(-0.0002~0.0014) | 0.147 |
| Q1 | 0(Ref) |  |  | 0(Ref) |  |
| Q2 | -0.0010 (-0.006~0.003) | 0.575 |  | 0.0011 (-0.0024~0.0047) | 0.537 |
| Q3 | -0.0030 (-0.0075~0.0014) | 0.178 |  | 0.0009(-0.0027~0.0044) | 0.637 |
| Q4 | -0.0046 (-0.009~-0.0002) | 0.042 |  | 0.0021 (-0.0015~0.0057) | 0.254 |
|  |  | 0.030 |  |  | 0.301 |
| **TBLH BMC z-Scores^**^** |  |  |  |  |  |
| DII | -0.0006(-0.0018~0.0007) | 0.363 |  | -0.0001(-0.001~0.0009) | 0.897 |
| Q1 | 0(Ref) |  |  | 0(Ref) |  |
| Q2 | 0.0033 (-0.0024~0.009) | 0.261 |  | 0.0007(-0.0035~0.005) | 0.741 |
| Q3 | 0.0014 (-0.0043~0.0071) | 0.630 |  | -0.0006(-0.005~0.0037) | 0.769 |
| Q4 | -0.003 (-0.0092~0.0023) | 0.239 |  | -0.0004(-0.0047~0.004) | 0.875 |
|  |  | 0.187 |  |  | 0.730 |

Crude model: no covariates were adjusted.

Adjusted model was adjusted for age, sex, race/ethnicity, education level, BMI, PIR, physical activity, white blood cell count, lymphocyte count, segmented neutrophils count, red cell distribution width, hemoglobin, platelet count, serum CRP, ALP, calcium, albumin, phosphorus, NHANES cycle.

DII, dietary inflammation index; BMAD_a_, Bone mineral apparent density for age; TBLH, total body less head; BMD, bone mineral density; BMC, bone mineral content; NHANES, National Health and Nutrition Examination Survey; BMI, body mass index; PIR, poverty income ratio; CI, confidence interval; Ref, reference; CRP, C-reactive protein; ALP, alkaline phosphatase. *data were obtained from the NHANES 2005-2010 cycles; **data were obtained from the NHANES 2001-2006 and 2011-2018 cycles.
